# Big data and its impact on the 3Rs: a home cage monitoring oriented review

**DOI:** 10.3389/fdata.2024.1390467

**Published:** 2024-05-20

**Authors:** Sara Fuochi, Mara Rigamonti, Eoin C. O'Connor, Paolo De Girolamo, Livia D'Angelo

**Affiliations:** ^1^Experimental Animal Center, University of Bern, Bern, Switzerland; ^2^Tecniplast S.p.A, Varese, Italy; ^3^Neuroscience and Rare Diseases, Roche Pharma Research and Early Development, Roche Innovation Center Basel, F. Hoffmann-La Roche Ltd., Basel, Switzerland; ^4^Department of Veterinary Medicine and Animal Production, University of Naples Federico II, Naples, Italy

**Keywords:** big data, home cage monitoring, refinement, reduction, replacement

## Abstract

Undisturbed home cage recording of mouse activity and behavior has received increasing attention in recent years. In parallel, several technologies have been developed in a bid to automate data collection and interpretation. Thanks to these expanding technologies, massive datasets can be recorded and saved in the long term, providing a wealth of information concerning animal wellbeing, clinical status, baseline activity, and subsequent deviations in case of experimental interventions. Such large datasets can also serve as a long-term reservoir of scientific data that can be reanalyzed and repurposed upon need. In this review, we present how the impact of Big Data deriving from home cage monitoring (HCM) data acquisition, particularly through Digital Ventilated Cages (DVCs), can support the application of the 3Rs by enhancing Refinement, Reduction, and even Replacement of research in animals.

## 1 Introduction

Big Data refers to extremely large and complex datasets that require complex data processing tools to secure storage and management, processing, and analysis. The basic characteristics of such datasets are outlined by the “Three Vs” of Big Data, originally described by Laney (2001). The concept of *Volume* refers to the vast amount of data contributing to the build-up of Big Data. This could range from terabytes to petabytes and beyond, but the sheer size of the dataset tends to be a key characteristic. The concept of *Velocity* typically refers to the fact that data—building up Big Data—is generated, collected, and processed at a very high speed. This can include data streaming in real-time from various sources, including sensors. The concept of *Variety* refers to the various formats encompassing a wide range of data types that are gathered to build Big Data. In addition to the original Three Vs, several authors and data scientists have considered or discarded many additional characteristics. For example, not all Big Data have volume, velocity, or variety (Kitchin and McArdle, 2016). Published datasets labeled as Big Data share many traits but vary in their characteristics and nature, acknowledging multiple forms of Big Data. Two of them, particularly contributing to the so-called “Five Vs” of Big Data, are worth mentioning. The first one is *Veracity*, referring to the quality of the data. Big Data can include data from unreliable sources or data with inconsistencies, making it crucial to assess and manage data quality. The last and certainly not the least one is *Value* (Günther et al., 2017). The ultimate goal of working with Big Data is to extract valuable insights, make better decisions, and create value for multiple stakeholders (Moorthy et al., 2015). The ability to turn large volumes of data into hands-on exploitable information is a key aspect.

The use of big data in animal research is becoming increasingly popular, allowing us to better understand animal behavior, health, and welfare. The amount of data generated by animal research is growing at an exponential rate, and the use of big data can help us make sense of this information to gain valuable insights. This is the case with the Digital Ventilated Cages (DVC^®^, Tecniplast S.p.A., Buguggiate VA, Italy) system, an automated home cage monitoring (HCM) technology that allows recording of mice activity 24/7, non-intrusively, and for an extended period. It consists of an electronic sensing board positioned below each cage position, composed of 12 contactless electrodes that measure electrical capacitance, which is impacted by the presence of animals. The board is connected via a wire to a power and data connection backbone infrastructure installed on the rack (Iannello, 2019). Each rack is then connected to a dedicated computer, which collects raw data from all the boards approximately 4 times per second (4 Hz). The raw data are first saved as zipped csv files, producing approximately 250 MB of data per day for a rack of 80 cage positions. All the raw data are then enriched with metadata cage information (such as cage IDs, mice IDs, and procedures on the cage), enabling automatic real-time position tracking for each cage within the facility and a massive Big Data collection from potentially thousands of cages simultaneously. This vast amount of data is uploaded to cloud storage to ensure data availability, integrity, and security, as well as fast and scalable data processing and algorithm calculation. Key metrics, such as locomotor activity, distance traveled, and index of bedding status, are calculated from the raw data in the cloud and then provided to a web-based software application (DVC^®^ Analytics, Tecniplast S.p.A., Buguggiate VA, Italy). Other metrics can then be derived from these (e.g., Golini et al., 2020; Fuochi et al., 2023) to generate novel *digital biomarkers* that can lead to new valuable insight into phenotypes and significantly help the application of the principles of the 3Rs (Baran et al., 2022).

## 2 Big data and the 3Rs

### 2.1 Refinement

The concept of *Refinement* is the amplest and most composite of the three Rs originally described by Russell and Burch (1959). In fact, *Refinement* has been described as the Cinderella of the 3Rs (Hau and Carver, 1994), progressively and constantly moving out from the shadow and gaining a primary role in enhancing animal wellbeing transversally to experimental design, husbandry, and procedural practices. Big Data deriving from HCM substantially contributed to the understanding of baseline undisturbed animal activity (Fuochi et al., 2021) and disturbed mouse behavior (Ulfhake et al., 2022). Most of all, Big Data can substantially contribute to the promotion of Refinement through 24/7 continuous recording, securing remote data accessibility and control, as well as high recording frequency, granting early detection of any change in motor behavior potentially suggestive of impairment of animal wellbeing. A study by Pernold et al. (2019) underscores Refinement by highlighting the implementation of a 24/7, scalable activity monitoring system that minimizes the impact on animals. In another study, Zentrich et al. (2021) clearly demonstrated how the DVC^®^ system could contribute to severity assessment as effectively as gold standard clinical parameters. These few examples already demonstrate the enormous potential for Big Data obtained through the DVC^®^ system to reduce stressors and enhance animal welfare. Refinement is evident in the ability to detect behavioral alterations, environmental influences, and even illness without subjecting animals to unnecessary handling or exposure to novel environments. Collectively, this can contribute to a more humane and improved experimental approach.

Another substantial contribution of Big Data deriving from HCM is the potential to detect, characterize, and implement biomarkers that can be used as early markers of disease progression. A remarkable example is a study from Golini et al. (2020), in which a non-intrusive assessment of sleep and rest disturbances is addressed in the SOD1G93A mouse model. Through Big Data analysis, a digital biomarker, the Regularity Disruption Index (RDI), is developed to quantify irregular activity patterns associated with amyotrophic lateral sclerosis progression. This non-intrusive approach allows continuous monitoring, contributing to the refinement of experimental methods by detecting subtle behavioral changes. The same analytic metric was employed to run an unobtrusive assessment of a novel rest-related phenotype in DMSXL mice, gaining valuable insights into potential mechanisms and therapeutic interventions for addressing excessive daytime sleepiness, enhancing the refinement of experimental methods, and contributing to the welfare of the mouse model in DM1 research (Golini et al., 2023). These examples highlight that the revolutionary aspect of Big Data is not only about the data itself but critically about analytical methods that can be developed to give new meaning to the data, in accordance with the principle expressed by King (2016).

### 2.2 Reduction

The R of reduction seeks to minimize the number of animals used in an experiment, to obtain information from fewer animals or more information from the same number of animals. Common methods to reduce animal numbers include power analysis, pilot studies, sharing data and resources (e.g., animals, tissues, and equipment) between research groups and organizations, appropriate experimental design, and adequate identification of control animals (Lee et al., 2022). The first intuitive impact of HCM on reducing the number of experimental animals is the longitudinal analysis, where quantitative measurements at time points common to each animal in the experiment are repeatedly recorded over time (“longitudinally”). Repeated measurements within the same animal can lead to a reduction in animal use, as compared to between-group designs, where each measurement is undertaken in different animals.

Importantly, in some longitudinal studies, each animal can serve as its own control at the baseline, further reducing the number of control animals (Kramer and Font, 2017). The core concept of DVC^®^ systems is to provide a minute-by-minute measure of undisturbed in-cage mouse activity, providing a vast amount of data favoring a granular phenotypic analysis. Interestingly, the highest volume of data generated may be used to draw a macroscopic analysis of behavioral activity, such as the reconstruction of a 24-h motor pattern (Fuochi et al., 2021) or to investigate the variety of motor responses at a specific moment in a mouse's life as determined by the researcher, i.e., motor activity following cage-change (Fuochi et al., 2021; Ulfhake et al., 2022) or comparing these measures among animal cages under the same standard conditions.

In addition to these examples, Big Data generated by automatic home cage recording systems has the potential to be reused for a completely different decision/task than for what it was originally intended (Woodall, 2017) and to describe new biological phenomena without the use of new animals (Fuochi et al., 2023). The availability of open-source data repositories, collecting data originating from *in vivo* research, can be accessed and used by researchers before planning new *in vivo* experiments. It allows for better orienting the scientific question and drastically reducing the total number of animals used in experiments, saving time, effort, and money and bringing research with animals within ethically acceptable bounds. Furthermore, several authors also highlight how including knowledge from historical experiments can contribute to limiting the number of animals used in a single experiment by recycling historical data (Walley et al., 2016; Kramer and Font, 2017). The possibility and reliability of this approach depend on the presence of suitable data from previously performed similar studies (Richter, 2024). In this regard, datasets derived from HCM can provide a valid and consistent source of recyclable knowledge thanks to its reproducibility-enhancing potential (Voikar and Gaburro, 2020).

### 2.3 Replacement

Replacement refers to the use of methods that avoid or substitute the use of animals in research (Russell and Burch, 1959). The concept of Replacement can be further divided into “*full”* or “*absolute”* Replacement, where non-animal methods are deployed in place of animal studies (Tannenbaum and Bennett, 2015). Alternatively, in “*partial”* or “*relative”* Replacement, non-animal studies are undertaken before moving into animal studies, or animals lower on the phylogenetic scale are given preference, such as rodents in place of non-human primates or the nematode *Caenorhabditis elegans* and the fruit fly *Drosophila melanogaster* in place of vertebrate species (Russell and Burch, 1959). Methods for Replacement include the use of biochemical assays and simple or more complex cellular model systems, such as organoids or organs-on-a-chip (Huang et al., 2021; Arjmand et al., 2023). Computer modeling and simulations (i.e., *in silico* methods) provide an increasingly important form of Replacement. Indeed, *in silico* methods for Replacement have increased rapidly in the past decade, thanks to the implementation of large, structured datasets and advanced analytical methods developed to interrogate them (Madden et al., 2020). Is there an opportunity for Replacement of rodents in HCM with alternative methods? At first glance, it would seem challenging, given that the primary focus of HCM is to record diurnal activity across the life of the laboratory rodent. Full replacement of this complex system with biochemical or *in vitro* methods is unlikely to ever be possible. On the other hand, the large quantities of structured data generated by HCM open the door toward *in silico* methods that could provide a Replacement function in two ways. First, HCM can contribute to Replacement through “data repurposing,” thus going beyond its Reduction potential. In this case, data generated in animals may be reused for a new purpose beyond its initial intended objective and without the need for further new animals. Opportunities for data repurposing can arise when new analysis methods and algorithms are developed and retroactively deployed on existing datasets to bring new insights (Fuochi et al., 2023).

A second scenario is the possibility of generating Virtual Control Groups (VCGs) in HCM experiments. VCGs are constructed from historical data and combined with computer simulations to generate control data that can be used as a full or partial replacement for new controls (Strayhorn, 2021). While most often deployed in a clinical research setting, VCGs are increasingly being explored for use in non-clinical research [for example, see the recently concluded IMI eTRANSAFE project (Steger-Hartmann et al., 2020, 2023)]. By replacing control group animals with existing randomized datasets, VCGs could reduce animal use by an estimated 25%. VCGs are also argued to support improvements in experiment repeatability and reproducibility, in part by ensuring transparent reporting of data (Moresis et al., 2024). The use of VCGs in non-clinical research is at an early stage, and the adoption of VCGs requires careful validation, considering both biological and statistical factors (Moresis et al., 2024). However, with a standardized approach to data capture and storage and highly scalable infrastructure, HCM represents an ideal context in which to further research and advance the use of VCGs.

## 3 Discussion

HCM systems are primarily defined by their ability to collect large amounts of data at a high sampling rate, long tracking duration, and scalability. Of note, the analysis of data from home-cage monitoring systems often requires the use of specific software tools to handle and process large datasets, especially when working on raw data and developing new tailored biomarkers. Some options include R and Python, two programming languages that offer a wide range of packages for data processing, statistical analysis, machine learning, and visualization. Moreover, the recent proliferation of AI chatbots such as OpenAI ChatGPT or Google Gemini opens possibilities for facilitating data analysis even without deep coding knowledge, both providing some basic data summary and offering scripts to assist users in coding. Additionally, future chatbots based on Large-language Models (LLMs) that can turn natural language requests into machine-executable code could be implemented for domain-specific and advanced analytical tasks (Ye et al., 2023).

In this review, we have outlined how Big Data generated by HCM can be structured and interpreted to meet the principles of Refinement, Reduction, and Replacement in animal research (Table 1).

**Table 1:** Interconnection between 5Vs, HCM data, and 3Rs.

**V**	**V in HCM data**	**R**	**Descriptive R**
**Volume**	High data volume recording. Multiple cages at the same time.	Refinement, Reduction, Replacement.	Punctual control of animal activity. Longitudinal studies with lower number of animals. Data repurposing.
**Velocity**	High-speed recording-−4 times per second (4 Hz).	Refinement.	Punctual control of animal activity. Potential to create early alerts with regard to animal welfare-related issues.
**Variety**	Board, Running Wheel, Food, Water, and RH in cage.	Refinement.	Multiparameter assessment of animal activity and behavior. Development of tailored metrics, early biomarkers, and endpoints.
**Veracity**	Direct raw data recording; integration possible with metadata from the cage.	Reproducibility, Reduction, and Refinement.	High reliability of data collected. Metrics development.
**Value**	Potential for data banks and share.	Replacement.	Perspective developments.

As for the *Refinement*, the non-intrusive but punctual control of animal activity, along with the development of tailored biomarkers, is an excellent achievement which, in addition to protecting the daily wellbeing of the animal, may assist in streamlining the management of animal facilities. The latter are often complex infrastructures, particularly when a plurality of mutant rodent models are housed, and thus, a differentiated and diversified welfare assessment must be adopted. The possibility of remotely evaluating the cage conditions (Ulfhake et al., 2022; Fuochi et al., 2023) and taking *ad hoc* actions is a further enhancement in the daily management of animal facilities.

As for Reduction, HCM can reduce the number of animals, thanks to the possibility of longitudinally recording animal activity by deploying repeated measures, within-animal study designs, and reducing the number of control arms. The digital recording of animal activity is a powerful method to maximize knowledge gain per animal used and repurpose the data to generate new knowledge. The data repurposing concept also fits well with *Replacement* (Fuochi et al., 2023). Data from HCM studies may enable the generation of virtual control groups or data repurposing and the support of meta-research. Critical to ensuring the success of these scenarios is that data collected from HCM studies is adequately described with metadata (i.e., information about the data). Metadata is essential for ensuring that raw or primary data stored in data repositories is FAIR (Findable, Accessible, Interoperable, Reusable) and enables researchers to interrogate data and understand the potential for repurposing. A “minimal metadata set” (referred to as “MNMS”) has recently been proposed to describe data generated from *in vivo* biomedical research experiments, with a view to enabling data repurposing (Moresis et al., 2024). To realize the full potential of HCM data to be used in scenarios of Replacement, as we have outlined, it will be essential that systems capture metadata in a way that minimizes the burden to the experimenter and that metadata follows raw/primary data into databases where legal considerations for data sharing have been established (Moresis et al., 2024). A key objective for Replacement is to increase the relevance and applicability of scientific studies to human health and disease by deploying more predictive models. This objective is driven by ethical considerations to reduce the use of animals in research, as well as scientific and regulatory demands to advance and adopt validated replacement methods (FDA report, 2021). The critical importance of *validation* in alternative methods (e.g., human cellular models or organs-on-a-chip) is also highly relevant for *in silico* methods that may be used in a Replacement context for HCM studies. Progress in other fields (Hasselgren et al., 2019) may inspire how frameworks for performing and reporting *in silico* assessments with HCM studies can ensure a transparent and consistent view of the results.

In conclusion, we have emphasized how data from HCM supports the 3Rs principle. It is mandatory to highlight, however, that what makes Big Data powerful is appropriate data analytical tools. Big Data only becomes qualitatively and quantitatively informative upon cleaning, aggregating, visualizing, and analyzing the data in a way that enables effective interpretation. The emerging fields of machine learning and data mining are instrumental in helping meet the challenges facing the analytics of HCM-derived data. It should also be highlighted that HCM-derived data can facilitate a change in the pre-clinical research mindset, shifting from a primarily hypothesis-driven to a data-driven approach. H-driven modeling brings a question into focus so that a model is constructed to investigate a specific hypothesis about how the system works or why certain phenomena are observed. Data-driven modeling, on the other hand, follows a more unbiased approach, with model construction informed by the computationally intensive use of data. Interestingly, Eriksson et al. (2022) recently proposed that data- and hypothesis-driven modeling approaches can be combined in neuroscience through a FAIR infrastructure. The workflows they have developed promise to increase the capacity for combining different types of models, extending models as new data accumulates, and validating existing models. If pursued in preclinical research settings, this kind of approach may lead to more robust, reliable, and reproducible data and further advance the 3Rs principle.

## Author contributions

SF: Conceptualization, Writing—original draft, Writing—review & editing, Supervision. MR: Conceptualization, Writing—original draft, Writing—review & editing. EO'C: Writing—original draft, Writing—review & editing. PD: Writing—review & editing. LD'A: Conceptualization, Supervision, Writing—original draft, Writing—review & editing.
